# Supplementary material for: Reconciling Mining with the Conservation of Cave Biodiversity: A Quantitative Baseline to Help Establish Conservation Priorities
Source: PLoS One. 2016 Dec 20;11(12):e0168348. doi: 10.1371/journal.pone.0168348 (PMC5173368; doi:10.1371/journal.pone.0168348)
Supplement: S1 Dataset — (ZIP) [file pone.0168348.s002.zip › Taxa/Serra Norte/SN_2013/N4WS/Lista N4WS-06.pdf]

## CAVIDADE N4WS-0006

| Classe | Ordem | Fam/Outros | Gên/Outros | Espécie | S-U | U-S | Zon. |
|---|---|---|---|---|---|---|---|
| Arachnida | Acari | Argasidae | *Ornithodoros* | sp.1 | X | X | P |
| Arachnida | Amblypygi | Charinidae | *Charinus* | sp.1 | 1 | 2 | E |
| Arachnida | Amblypygi | Phrynidae | *Heterophrynus* | *longicornis* | 1 | | E |
| Arachnida | Araneae | Araneidae | *Alpaida* | *septemmammata* | X | | E |
| Arachnida | Araneae | Corinnidae | *Creugas* | sp.1 | | 1 | P |
| Arachnida | Araneae | Filistatidae | | sp.1 | | X | E |
| Arachnida | Araneae | Ochyroceratidae | *Speocera* | sp.1 | X | X | P |
| Arachnida | Araneae | Pholcidae | *Mesabolivar* | *aurantiacus* | | X | E |
| Arachnida | Araneae | Pholcidae | *Mesabolivar* | *eberhard* | X | X | P |
| Arachnida | Araneae | Salticidae | | jovem | X | | E |
| Arachnida | Araneae | Scytodidae | *Scytodes* | *globula* | 7 | 8 | E/P |
| Arachnida | Araneae | Theraphosidae | aff. *Holothele* | sp.1 | 12 | 1 | E |
| Arachnida | Araneae | Theridiosomatidae | *Plato* | sp.1 | X | X | E/P |
| Arachnida | Araneae | Uloboridae | *Uloborus* | sp.2 | X | | E |
| Arachnida | Opiliones | Sclerosomatidae | | jovem | | X | P |
| Arachnida | Palpigradi | Eukoeneniidae | *Leptokoenenia* | *thalassophobica* | | X | P |
| Arachnida | Pseudoscorpiones | Chernetidae | *Spelaeochernes* | sp.1 | X | X | E/P |
| Arachnida | Pseudoscorpiones | Chthoniidae | *Pseudochthonius* | sp.1 | | X | E |
| Diplopoda | Spirostreptida | Pseudonannolenidae | *Pseudonannolene* | sp.1 | | 2 | P |
| Entognatha | Collembola | Paronellidae | | sp.4 | X | | P |
| Entognatha | Diplura | Campodeidae | | sp.1 | | X | P |
| Entognatha | Diplura | Japygidae | | sp.1 | | X | P |
| Entognatha | Diplura | Projapygidae | | sp.1 | | X | E |
| Insecta | Blattodea | | | jovem | 1 | | P |
| Insecta | Diptera | Cecidomyiidae | *Asphondylia* | sp. | X | | P |
| Insecta | Diptera | Ceratopogonidae | | sp. | X | | P |
| Insecta | Diptera | Clusiidae | | sp. | X | | E |
| Insecta | Diptera | Culicidae | *Culex* | sp. | | X | E |
| Insecta | Diptera | Dolichopodidae | | sp. | X | | E |
| Insecta | Diptera | Ephydridae | | sp. | X | | E |
| Insecta | Diptera | Muscidae | | sp. | X | | E |
| Insecta | Diptera | Mycetophilidae | *Neoempheria* | sp. | | X | E |
| Insecta | Diptera | Psychodidae | *Sciopemyia* | *sordellii* | X | | P |
| Insecta | Diptera | Sciaridae | *Pseudosciara* | sp. | X | | P |
| Insecta | Diptera | Tachinidae | Exoristinae | sp. | X | | E |
| Insecta | Diptera | Tipulidae | | jovem | X | | E |
| Insecta | Heteroptera | Lygaeidae | Rhyparochrominae | sp.1 | X | X | E |
| Insecta | Heteroptera | Miridae | | sp.1 | X | | E |
| Insecta | Heteroptera | Reduviidae | Reduviinae | jovem | 3 | 3 | E/P |
| Insecta | Heteroptera | Schizopteridae | Schizopterinae | sp.1 | | X | E |
| Insecta | Hymenoptera | Apoidea | | sp.1 | 2 | | E |
| Insecta | Hymenoptera | Braconidae | | sp.1 | X | | E |
| Insecta | Hymenoptera | Formicidae | *Brachymyrmex* | sp.1 | X | | E |
| Insecta | Hymenoptera | Formicidae | *Camponotus* | sp.1 | X | | E |
| Insecta | Hymenoptera | Formicidae | *Dolichoderus* | aff. *bispinosus* | | X | E |
| Insecta | Hymenoptera | Formicidae | *Hypoponera* | sp.1 | X | | P |
| Insecta | Hymenoptera | Formicidae | *Odontomachus* | sp.1 | 2 | 1 | P |
| Insecta | Hymenoptera | Formicidae | *Pachycondyla* | sp.1 | X | | P |
| Insecta | Hymenoptera | Formicidae | *Solenopsis* | sp.1 | X | | P |

| | | | | | | | |
|---|---|---|---|---|---|---|---|
| Insecta | Hymenoptera | Formicidae | *Solenopsis* | sp.2 | | X | P |
| Insecta | Isoptera | Termitidae | *Nasutitermes* | sp. | X | X | E |
| Insecta | Lepidoptera | Glossata | | sp.1 | X | | E |
| Insecta | Lepidoptera | Noctuoidea | | sp.12 | 3 | | E |
| Insecta | Lepidoptera | Tineoidea | | sp.6 | | X | P |
| Insecta | Neuroptera | Myrmeleontidae | | jovem | 5 | 7 | E/P |
| Insecta | Orthoptera | Phalangopsidae | *Phalangopsis* | sp. | 7 | 18 | E/P |
| Insecta | Psocoptera | Epipsocidae | *Epipsocus* | sp.2 | | X | E |
| Insecta | Psocoptera | Psyllipsocidae | *Psocathropos* | sp.1 | | X | E |
| Insecta | Thysanoptera | Thripidae | | sp.1 | | X | E/P |
| Insecta | Thysanura | Nicoletiidae | | jovem | X | | P |
| Gastropoda | Pulmonata | Subulinidae | *Lamellaxis* | sp. | | X | E |
| Amphibia | Anura | Strabomantidae | *Pristimantis* | cf. *fenestratus* | 1 | | E |
| Ave | | | | sp. | 2 | | E |
| Mammalia | Chiroptera | Emballonuridae | *Peropteryx* | sp. | 10 | 5 | E/P |
| Mammalia | Rodentia | Cricetidae | *Rhipidomys* | sp. | 1 | | E |
